# Evolutionary Assembly and Future Design of Gibberellin Signaling

**DOI:** 10.1002/advs.75049

**Published:** 2026-03-28

**Authors:** Weishu Fan, Xihan Chen, Dan Li, Xiuhua Gao, Xiangdong Fu

**Affiliations:** ^1^ State Key Laboratory of Seed Innovation Institute of Genetics and Developmental Biology Chinese Academy of Sciences Beijing China; ^2^ School of Life Sciences Yunnan University Kunming China; ^3^ New Cornerstone Science Laboratory College of Life Sciences University of Chinese Academy of Sciences Beijing China

**Keywords:** AI‐driven crop design, directed evolution, Gibberellin signaling, nitrogen use efficiency

## Abstract

Gibberellin (GA) signaling is widely regarded as a canonical hormone‐controlled growth pathway, yet accumulating evidence suggests that it emerged through stepwise evolutionary assembly rather than as a pre‐formed regulatory system. Here, we revisit the origin and diversification of the GA pathway from an evolutionary perspective. DELLA‐mediated growth repression arose during early land plant evolution and was later integrated into a hormone‐responsive framework following the establishment of GA biosynthesis and perception. The emergence of the receptor GID1 completed the core GA–GID1–DELLA module, enabling dynamic hormonal control of plant growth. Subsequent diversification of this module underpinned key agricultural innovations, including the semi‐dwarf traits of the Green Revolution. However, manipulation of GA signaling also exposed trade‐offs, particularly between reduced plant height and nitrogen use efficiency (NUE). Recent advances reveal additional regulatory nodes linking GA signaling to metabolic and stress‐responsive networks, highlighting the importance of precise hormonal fine‐tuning. Looking forward, we propose that integrating comparative genomics, structural biology, protein language models, and artificial intelligence may enable rational rewiring of GA signaling, providing a blueprint for engineering climate‐resilient and resource‐efficient crops.

Gibberellin (GA) signaling, usually presented as a textbook example of a hormone‐controlled growth pathway, is better understood as a product of stepwise evolutionary assembly rather than a pre‐formed regulatory system. Comparative genomic, biochemical, and functional evidence increasingly suggests that the capacity to synthesize and perceive bioactive GAs arose after plants had already established terrestrial lifestyles, with a fully hormone‐responsive GA signaling system becoming fixed in vascular plant lineages. From facilitating adaptive growth in early land plants to shaping the semi‐dwarf architectures that underpinned the Green Revolution, the GA pathway has repeatedly been modified by evolution and human selection. In the current era of genomic‐scale data, high‐resolution protein structures, and rapidly advancing artificial intelligence (AI), GA signaling has become a powerful model for understanding how complex signaling systems evolve and how evolutionary constraints can be exploited to design the next generation of crop traits (Figure 1).

**FIGURE 1:**
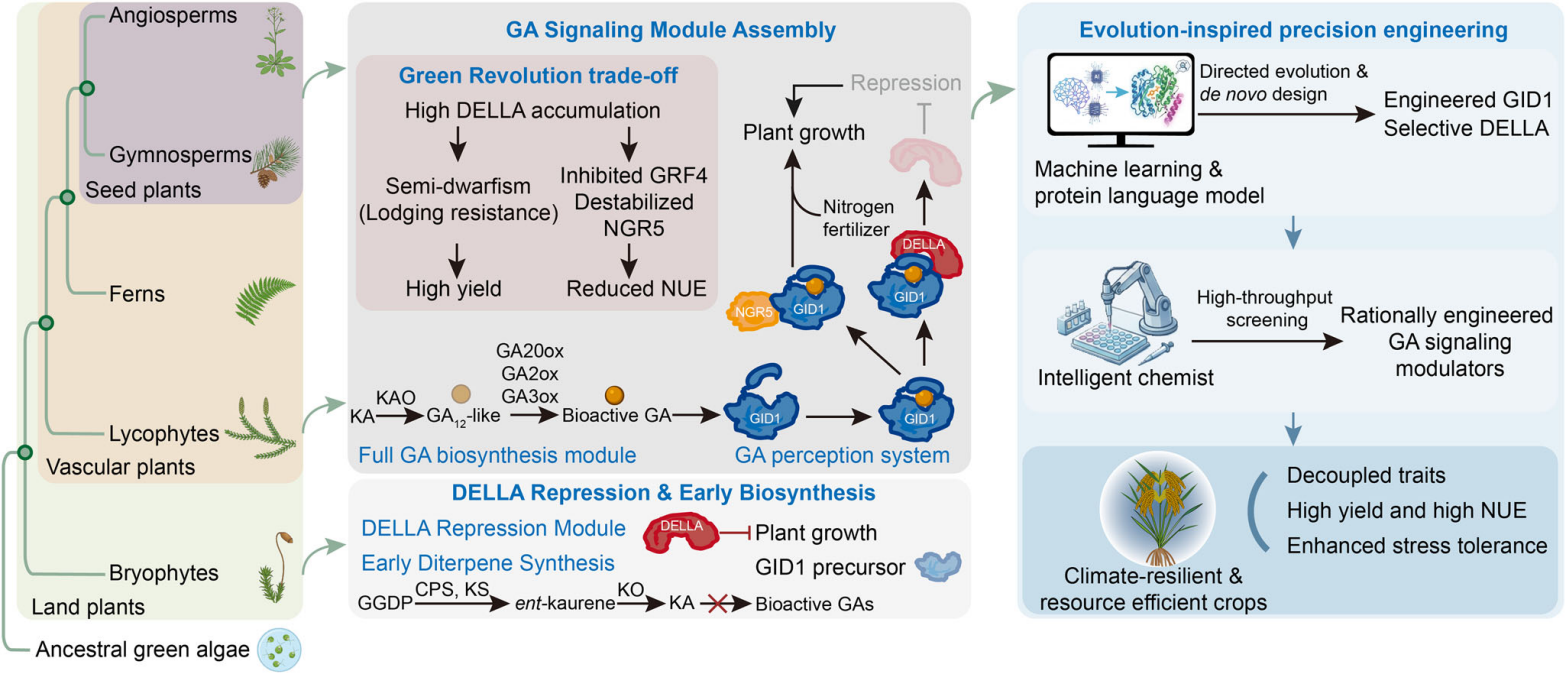
Evolutionary trajectory of gibberellin signaling and its rational design for climate‐resilient and resource efficient crops. This schematic illustrates the stepwise evolutionary assembly of the GA signaling pathway (left panel), its core functional modules and the agronomic trade‐off highlighted by the Green Revolution (center panel), and the emerging strategies for its precision engineering to improve crops (right panel). The pathway evolved from an ancestral DELLA‐mediated growth repression system in early land plants, with subsequent acquisition of GA biosynthesis and the GID1 receptor. Understanding of this assembly, particularly the trade‐off between semi‐dwarfism and NUE, now informs AI‐driven and synthetic biology approaches, such as *de novo* protein design and rationally engineered GA signaling modulators, to rewire the pathway for climate‐resilient, high‐yield crops.

Growth repression mediated by DELLA proteins predates the emergence of GA perception and was later incorporated into the GA signaling framework during land plant evolution. Comparative genomic analyses reveal that *DELLA* genes are conserved across all examined land plant lineages, including bryophytes and vascular plants, but are absent from algae, indicating that DELLA‐mediated growth inhibition originated during terrestrialization [1]. Functional conservation across deep evolutionary distances further supports this interpretation: DELLA proteins from mosses and lycophytes retain robust growth‐repressive activity when expressed in flowering plants, demonstrating that their core biological role predates the establishment of canonical hormone‐dependent regulation [2]. Subsequent evolution of GA signaling therefore did not invent growth repression *de novo*, but instead recruited and refined this pre‐existing regulatory module, conferring hormonal control onto an already established growth‐inhibitory framework.

The emergence of GA signaling required not only regulatory innovation but also the stepwise evolution of a dedicated biosynthetic machinery capable of generating bioactive GAs. The early steps of GA biosynthesis, catalyzed by *ent*‐copalyl diphosphate synthase (CPS) and *ent*‐kaurene synthase (KS), trace back to an ancestral bifunctional CPSKS enzyme in early land plants [3]. Phylogenetic and biochemical evidence indicate that this ancestral CPSKS produced *ent‐*kaurene for phytohormone biosynthesis and that subsequent gene duplication and neofunctionalization events gave rise to the diverse terpene synthase (TPS) lineages involved in specialized diterpenoid metabolism. In non‐vascular plants, these enzymes typically exist as multifunctional or multicopy forms, maintaining the conserved production of *ent‐*kaurene for phytohormone biosynthesis while allowing paralogous lineages to diversify for lineage‐specific diterpene metabolites. Importantly, bryophytes retain *ent*‐kaurene production and commonly accumulate *ent‐*kaurenoic acid (KA), representing a conserved ancestral metabolic intermediate with no evidence of a fully elaborated GA biosynthetic pathway. Subsequent oxidation of *ent*‐kaurene by cytochrome P450 (CYP) mono‐oxygenases, e.g., *ent*‐kaurene oxidase (KO), leads to the formation of KA. Genes encoding *ent*‐kaurenoic acid oxidase (KAO), which mediates the next oxidative step toward GA_12_ [4], were absent in extant moss lineages, which may contribute to the accumulation of KA as a predominant terminal metabolite in those taxa. A major evolutionary transition occurred with the appearance of the 2‐oxoglutarate‐dependent dioxygenases (2‐OGD) family, including GA20ox, GA3ox, and GA2ox, which form a complete pathway only in vascular plants. This enzymatic refinement established the biochemical prerequisites for hormone‐dependent growth regulation and set the stage for the subsequent evolution of GA perception mechanisms.

Once plants evolved the capacity to produce bioactive GAs in a controlled manner, perception of these signals became an essential next step. Following early hypotheses of membrane‐bound receptors, the discovery of GIBBERELLIN INSENSITIVE DWARF1 (GID1) in rice established a new paradigm for GA perception as a soluble receptor [5]. Structural studies later revealed that GID1 evolved from the hormone‐sensitive lipase (HSL) family within the α/β‐hydrolase superfamily, transitioning from a catalytic enzyme into a specialized receptor through loss of hydrolase activity and remodeling of its active site into a high‐affinity GA‐binding pocket [6]. GA serves as an allosteric inducer: its binding triggers the closure of a flexible N‐terminal “lid”, creating a hydrophobic interface that facilitates the recruitment of the DELLA repressor. This GA‐GID1‐DELLA signaling module is highly conserved and functional across vascular plants. In angiosperms, gene duplication and subfunctionalization further expanded this module, giving rise to receptor variants with distinct expression patterns and sensitivities. This regulatory plasticity allowed flowering plants to integrate complex developmental programs with fluctuating environmental cues. Building on this regulatory plasticity, the GA‐GID1‐DELLA module is now widely recognized as a central regulatory hub that integrates growth control with environmental and metabolic signals. A key extension of this canonical module is provided by NGR5, an AP2‐domain transcription factor that acts as a direct ubiquitination substrate for GID1 independently of DELLA, thereby coupling GA signaling with nitrogen‐responsive chromatin regulation [7]. In parallel, GRF4 was identified as a positive regulator that directly promotes the expression of genes involved in carbon and nitrogen uptake [8]. While the Green Revolution successfully exploited DELLA accumulation to improve lodging resistance, this strategy physically inhibits the GRF4‐mediated metabolic hub and promotes NGR5 instability, leading to declined nitrogen use efficiency (NUE) and increased dependency on chemical fertilizers. Recent work further extends this framework by showing that precise GA fine‐tuning, rather than simple pathway activation or repression, is essential for abiotic stress adaptation, as it balances NGR5‐mediated epigenetic repression of stress‐tolerance genes with the deleterious effects of reactive oxygen species‐induced toxicity under thermal and alkali stress [9].

From the metabolic exploration of ancestral land plants to the agronomic optimization of modern crops, the evolutionary logic of the GA signaling pathway provides a precious genetic blueprint. This perspective opens new opportunities for rational pathway rewiring beyond traditional gain‐ or loss‐of‐function approaches. Recent advances in artificial intelligence (AI)‐driven protein design now enable predictive exploration of protein sequence space. High‐accuracy structure prediction tools such as AlphaFold and multimer models allow reliable modeling of receptor‐regulator complexes [10], while protein language models (e.g., ESM) can propose functionally plausible mutations without explicit structural templates [11]. Generative approaches, including RFdiffusion and ProteinMPNN, further permit backbone generation or sequence optimization under defined structural constraints [12, 13]. These methods have already delivered experimental successes, including language model‐guided antibody affinity maturation, AI‐assisted viral capsid optimization from vast virtual libraries, and *de novo* luciferases with exceptional thermal stability [14]. Specifically, recent studies have reported the successful computational *de novo* design of functional serine hydrolases exhibiting measurable catalytic activity, marking a significant advance in AI‐enabled enzyme engineering [15]. Additionally, structure‐based clustering of protein architectures has revealed previously unrecognized deaminase functions, illustrating the power of structural AI approaches for functional discovery [16].

By integrating large‐scale comparative genomics, protein language models, high‐resolution structural data, and AI, it should become feasible to pursue the *de novo* design and directed evolution of GA signaling components, enabling targeted uncoupling of desirable traits such as semi‐dwarfism from penalties in NUE or stress tolerance. By collaborating with AI‐driven chemical platforms, which combine machine learning with high‐throughput chemical synthesis, researchers can now explore an expanded chemical space for synthetic GA analogs with heightened specificity for particular receptor variants. Furthermore, the integration of AI‐driven precision engineering allows for the “rewiring” of the GA perception module; for instance, by modifying the GID1‐NGR5 interaction interface to stabilize NGR5 under variable GA conditions, or by engineering DELLA variants that selectively decouple height control from the repression of GRF4‐mediated metabolism. Such interdisciplinary approaches move beyond traditional breeding and random mutagenesis. More broadly, GA signaling could serve as a prototype for AI‐driven reconstruction of plant hormone networks, where evolutionary constraints are not merely descriptive but become actionable design principles for engineering climate‐resilient, resource‐efficient crops.

## Funding

This work was supported by grants from the National Natural Science Foundation of China (32588101), the Youth Innovation Promotion Association CAS (2023408), the National Natural Science Foundation of China (32370282), and the New Cornerstone Investigation Program (NCI202234).

## Conflicts of Interest

The authors declare no conflicts of interest.

## Data Availability

Data sharing not applicable to this article as no datasets were generated or analysed during the current study.
